# Obituary

**Published:** 1830

**Authors:** 


6. *Obituary.*—Died at his residence near Philadelphia, on the 17th April, Dr. JOHN D. GODMAN, late Professor of Anatomy in the Rutgers Medical College of New-York. A sketch of the life and character of this talented and amiable man, will be given in our next number. D.

